# Physicochemical indication of the FLASH effect from shoot-through proton pencil beam scanning parameters delivered under ultra-high dose rates

**DOI:** 10.1088/1361-6560/adf58e

**Published:** 2025-08-19

**Authors:** Andrew M Friberg, Hai Siong Tan, Eric S Diffenderfer, Ioannis Verginadis, Michele M Kim, Keith Cengel, Rodney Wiersma, Lei Dong, Costas Koumenis, Boon-Keng K Teo, Wei Zou

**Affiliations:** 1 Department of Radiation Oncology, University of Pennsylvania, Philadelphia, PA, United States of America; 2 Department of Radiation Oncology, UCLA, Los Angeles, CA, United States of America

**Keywords:** FLASH, UHDR, pencil beam scanning, peroxyl radicals, modeling

## Abstract

*Objective.* Ultra-high dose rate (UHDR) proton pencil beam scanning (PBS) delivery results in irregular temporal-varying dose accumulation. It is difficult to establish a dose rate standard for the indication of proton PBS FLASH effect. In this work, we adopted a published physicochemical approach and investigated the impact of proton PBS UHDR parameters on the formation and downstream reactions of reactive oxygen species (ROS). *Approach.* From the ROS physicochemical model, the dose-rate dependent alkyl hydroperoxide (ROOH) formation was validated against published lipid peroxide absorbance data and correlated with mice skin damage data. For proton PBS delivery with specified beam current, voxelized temporal dose and ROS accumulation was calculated at the plateau region to simulate a shoot-through FLASH delivery. The ROS were obtained mimicking the irradiation of hypoxic skin. We examine the ROS-volume histogram in relation to the proton PBS delivery parameters. *Main results.* ROOH production clearly indicates sparing effects under UHDR. For PBS deliveries of 10 Gy to a 100 × 100 mm^2^ field at 8 mm depth, the ROOH yield at 500 nA FLASH beam current is equivalent to a 8.78 Gy delivery at 1nA CONV delivery. The yield of ROOH depends strongly on the dose and beam current but has minimal dependency on the field size and spot spacing. Introducing inter-beam intervals of two minutes reduces the FLASH reduction in ROOH, consistent with reduced FLASH effect in murine experiment. *Significance.* The volumetric statistics of the ROOH yield showed consistent indication of FLASH effects in preclinical observations and correlated with the lipid peroxidation damage in tissue. Using simulated ROOH production metrics can potentially indicate the FLASH sparing effect under various PBS delivery parameters. Our simulations indicate that the shoot-through PBS FLASH effect depends mainly on the total dose and the pencil beam current, and is relatively independent of field sizes and spot spacings.

## 1. Introduction

Ultra-high dose rate (UHDR) radiotherapy has been shown to spare normal tissue while maintaining tumor control efficacy. This FLASH effect has been demonstrated in numerous pre-clinical studies in electron, proton, photon, carbon ion, and helium ion modalities (Favaudon *et al* 2014, Montay-Gruel *et al* 2018, 2019, Diffenderfer *et al* 2020, Zhang *et al* 2020, Gao *et al* 2022, Tinganelli *et al* 2022). It may also provide benefits in challenging re-irradiation settings, even when the first delivery was at conventional dose rates (Dai *et al* 2023, Verginadis *et al* 2024). While it is common to cite a mean dose rate of 40 Gy s^−1^ or above as the threshold for FLASH, some authors have pointed out that related delivery parameters such as the fractional dose, instantaneous dose rate and mean dose rate may be important for UHDR irradiations (Favaudon *et al* 2014, Montay-Gruel *et al* 2021, Sunnerberg *et al* 2023, Loo *et al* 2024).

Most of the FLASH preclinical studies with protons have relied on pencil beams accelerated by isochronous cyclotrons (Diffenderfer *et al* 2020, Zhang *et al* 2020, Gao *et al* 2022, Sørensen *et al* 2022). For scattered field, isochronous cyclotron generates a single pulse of quasi-continuous dose in small animals positioned at the beam entrance plateau region, with dose rates that can be tuned from conventional (CONV) $\sim1\,{\text{Gy}}\,{{\text{s}}^{ - 1}}$ to UHDR levels $\sim100\,{\text{Gy}}\,{{\text{s}}^{ - 1}}$, using $225\,{\text{MeV}}$ proton pencil beam current greater than ${ }200\,{\text{nA}}$ just upstream of the scatterer (Zou *et al* 2020). Recently our center has achieved a stable FLASH configuration with $500\,{\text{nA}}$ pencil beam current at the nozzle in a gantry room for FLASH pencil beam scanning (PBS) delivery.

The potential benefits of FLASH have prompted the start of clinical trials. A patient with cutaneous lymphoma treated with UHDR electrons demonstrated comparable acute and late effects and tumor control to a CONV electron treatment (Gaide *et al* 2022). Two electron FLASH trials, one for metastatic skin melanoma patients and one for localized cutaneous basal cell carcinoma or squamous cell carcinoma, were started in 2021 (IMPulse, ClinicalTrials.gov no. NCT04986696) and 2023 (LANCE, ClinicalTrials.gov no. NCT05724875). In US, the FAST-01 clinical trial demonstrated its feasibility in delivering proton FLASH-RT and tumor control in patients with bone metastasis (Mascia *et al* 2023a). The FAST-02 proton FLASH-RT trial for bone metastases in the thoracic region is now ongoing (ClinicalTrials.gov no. NCT05524064).

Proton FLASH irradiation of larger tumors in human patients requires PBS setup. FLASH planning studies and clinical trials using PBS to deliver an array of pencil beam spots to cover field with lateral size of a few centimeters or larger (van de Water *et al* 2019, van Marlen *et al* 2020, Kang *et al* 2022, Lee *et al* 2022). To achieve the highest dose rates the proton accelerator was tuned to the highest achievable pencil beam current, in comparison with conventional PBS treatments which use a few nA at the nozzle level (Darafsheh *et al* 2020, Diffenderfer *et al* 2020, Titt *et al* 2022). However, the definition of dose rate is not as straightforward in PBS as compared to non-scanned scattered irradiation in preclinical studies. For instance, quasi-continuous cyclotron generated PBS plans result in each treated voxel receiving a unique temporal dose structure, with contributions from multiple spots. This non-homogenous dose-time structure is further complicated by the pulsed beam generation from synchrocyclotrons, or the spill structure from synchrotrons (Darafsheh *et al* 2020, Titt *et al* 2022). To account for the complicated FLASH PBS delivery, various dose rate definitions have been proposed: average dose rate (ADR) uses the voxel dose divided by the field delivery time (Zou *et al* 2020, Mascia *et al* 2023a); dose-averaged dose rate (DADR) emphasizes the importance of the spot dose to the local dose rate (van de Water *et al* 2019); PBS-dose rate (PBSDR) discounts the delivery time spent on spots that are distant from local voxels and thus contribute minimal dose (Folkerts *et al* 2020). However, these definitions can generate FLASH dose rate values that differ by multiple orders of magnitude therefore making the PBSDR impossible to compare to the canonical UHDR threshold developed in preclinical study, e.g. $40\,{\text{Gy}}\,{{\text{s}}^{ - 1}}$. In addition, the impact of detailed dose-time structure has not been explored owing to a lack of biological outcome data (Deffet *et al* 2023).

The mechanism of FLASH sparing is under active investigation, and several hypotheses have been put forward (Wilson *et al* 2020, Limoli and Vozenin 2023). One of the earliest was the suggestion that near-hypoxic tissue experiences oxygen reduction under UHDR leading to transient hypoxia that protects the tissue from further radiation damage (Pratx and Kapp 2019a, 2019b, Zou *et al* 2022). There are some biological indicators that FLASH has reduced inflammation, which could explain reduced damage post-irradiation and points to a reduction of pro-inflammatory markers (Kim *et al* 2024, Ni *et al* 2025). Another popular hypothesis suggests reduced oxidative stress due to rapid radical–radical recombination under UHDR that leads to reduced inflammation and cell damage (Limoli and Vozenin 2023, Grilj *et al* 2024). Studies in various assays indicated reduced hydrogen peroxide under UHDR conditions (Montay-Gruel *et al* 2019, Kacem *et al* 2022, Froidevaux *et al* 2023, Sunnerberg *et al* 2023, Thomas *et al* 2023). Recent experiments indicate that FLASH irradiated cell membrane model has much reduced lipid peroxidation compared to CONV, which can be due to increased bi-molecular recombination of ${\text{L}} \cdot $ at high dose rates (Froidevaux *et al* 2023, Grilj *et al* 2024).

The radical–radical combination processes can be simulated. The radiation physics and physicochemical simulations for water radiolysis and oxidative damage of the DNA substrates were carried out with Monte Carlo simulations, which have excellent temporal and spatial resolution (Incerti *et al* 2016, Schuemann *et al* 2019, Lai *et al* 2020, Ramos-Méndez *et al* 2020, Shin *et al* 2024). These studies have shown temporal dynamics of radiolytic products over the microsecond time scale at which point diffusion will have ensured a rather uniform concentration. These conditions are amenable to chemical modeling using reaction constants which efficiently accounts for radiochemical dynamics among various species of much larger quantities and over a much longer period of time (Labarbe *et al* 2020). Such simulations have suggested that the production of reactive oxygen species (ROS) is highly dose and dose rate dependent that are relevant in FLASH studies (Labarbe *et al* 2020, Tan *et al* 2023).

In this study, we extended our previous work with physicochemical simulations (Tan *et al* 2023) to the realm of proton PBS scanning, using the ROS product to indicate the FLASH effect under UHDR PBS delivery, in lieu of using one of the proposed dose rate definitions. We first demonstrated the agreement between our simulation of alky peroxide (ROOH) yield under different electron dose rates and the recently published lipid peroxide spectrophotometer measurement (Froidevaux *et al* 2023). The ROOH yields also indicated the murine skin damage under scattered CONV and FLASH proton irradiations. We then applied the model for ROS production under various shoot-through proton PBS delivery conditions to examine the impact of total dose, PBS dose temporal structure due to spot current, spacing, and field size. We introduced the ROS-volume histogram (RVH), in particular, ROOH-volume histogram as a metric for the PBS delivery to a defined region and assessed its correlation with various dose rate definitions. Additionally, the impact of time intervals on the ROOH yield during multi-beam isodose deliveries was simulated and compared to the preclinical observations (Mascia *et al* 2023b).

## 2. Methods

### 2.1. Physicochemical model and tissue conditions

We modified the model of Labarbe *et al* based on Wardman and our earlier work (Labarbe *et al* 2020, Wardman 2020, Tan *et al* 2023). Twelve dynamical species were incorporated, specifically aqueous electrons ${\text{e}}_{{\text{aq}}}^ - $, hydrogen radicals ${{\text{H}}^ \cdot }$, hydroxyl radicals ${\text{O}}{{\text{H}}^ \cdot }$, hydrogen gas ${{\text{H}}_2}$, hydrogen peroxide ${{\text{H}}_2}{{\text{O}}_2}$, superoxide ${\text{O}}_2^ \cdot $, hydroperoxyl ${\text{HO}}_2^ - $, radical scavenger such as intra-cellular thiol ${\text{GSH}}$ and its radical form ${\text{GS}} \cdot $ $,$ alkyl radicals ${{\text{R}}^ \cdot }$, alkyl peroxyl radicals ${\text{RO}}{{\text{O}}^ \cdot }$, and alkyl peroxide ${\text{ROOH}}$. The model also involves the local oxygen ${{\text{O}}_2}$, abundant water ${{\text{H}}_2}{\text{O}}$ content and generic carbon-based biomolecules ${\text{RH}}$. The concentration of each species $\left[ {{M_i}} \right]$ was formulated as a system of 12 non-linear coupled ordinary differential equations, generally of the form

\begin{equation*}\frac{{{\text{d}}\left[ {{M_i}} \right]}}{{{\text{d}}t}} = {G_i}{\rho _{\text{w}}}\dot D\left( t \right) + \mathop \sum \limits_{j = 1,{ }j \ne i}^{12} \mathop \sum \limits_{k = j,{ }k \ne i}^{12} C_i^{jk}\left[ {{M_j}} \right]\left[ {{M_k}} \right] - \mathop \sum \limits_{k = 1,{ }k \ne i}^{12} C_i^{ik}\left[ {{M_i}} \right]\left[ {{M_k}} \right]\end{equation*}

where ${G_i}$ is the primary yield due to radiation, ${\rho _{\text{w}}}$ is the density of water, $\dot D\left( t \right)$ is the local instantaneous dose rate, and $C_i^{jk}$ are the rate constants for reactions between species $j$ and $k$. The first term describes the initial radiolysis of water under irradiation conditions, which leads to the production of ${\text{e}}_{{\text{aq}}}^ - $, ${\text{O}}{{\text{H}}^ \cdot }$, ${{\text{H}}^ \cdot }$, ${{\text{H}}_2}$, and ${{\text{H}}_2}{{\text{O}}_2}$. Radiation with low linear energy transfer (LET) produces disperse ‘spurs’ of ROS products, whereas higher LET radiation causes increases in the local concentration of ROS and consequently changes the number of radicals escaping from these tracks. We account for these differences in beam type and quality by changing the initial primary yields and simulate successive reactions according to the rate constants $C_i^{jk}$ (table 1).

**Table 1.** Primary yields used in the simulation for 6 MeV electron beams and in the plateau region of 227 MeV proton beams. The yield values are given in units of *μ* mol J^−1^.

Radical species	6 MeV electrons	Protons (227 MeV, entrance)
${\text{e}} \cdot $	0.280	0.260
${\text{OH}} \cdot $	0.280	0.270
${\text{H}} \cdot $	0.0580	0.0586
${{\text{H}}_2}$	0.0456	0.0504
${{\text{H}}_2}{{\text{O}}_2}$	0.0746	0.0698

Radicals resulting from the initial irradiation propagate and react with organic substrates RH leading to radiation induced degradation of nucleic acid and lipids. The concentration of RH is set to be 1 M to reflect its significantly larger concentration than that of other radicals in the tissue environment. Abstraction of hydrogen from RH substrate molecules leads to radicals ${\text{R}} \cdot $ which natural decay with rate constant 300 ${{\text{s}}^{ - 1}}$ or self-react to form dimers. In the presence of oxygen, it is possible to form peroxyl radicals ${\text{ROO}} \cdot $ which themselves decay with rate constant 0.6908 ${{\text{s}}^{ - 1}}$. The decays of ${\text{R}} \cdot $ and ${\text{ROO}} \cdot $ were built in our model (table 2) although not indicated explicitly in the mathematical expression of equation (1). The process of radical scavenging is critical to cellular homeostasis to protect against damaging radicals through several bio-mechanisms (Wardman 2020). Many of these pathways rely heavily on the cysteine-containing tripeptide glutathione (GSH). Our model also tracks the degradation of GSH to ${\text{GS}} \cdot $ following their radical reduction of ${{\text{R}}^ \cdot }$ and ${\text{RO}}{{\text{O}}^ \cdot }$. The reaction constants and their related reactions used in our model are listed in table 2.

**Table 2.** The reaction constants and their related reactions used in our physicochemical model.

Rate constant	Numerical value (ms^−1^)	Reaction
$C_1^{1w},{\text{ }}C_5^{1w}$	$1.9 \times 10$	${{\text{e}}^ - } + {{\text{H}}_2}{\text{O}} \to {\text{H}} \cdot + {\text{O}}{{\text{H}}^ - }$
$C_1^{11},{\text{ }}C_6^{11}$	$1.1 \times {10^{10}}$	${{\text{e}}^ - } + {{\text{e}}^ - } \to {{\text{H}}_2} + 2{\text{O}}{{\text{H}}^ - }$
$C_1^{15},{\text{ }}C_5^{15},{\text{ }}C_6^{15}$	$2.5 \times {10^{10}}$	${{\text{e}}^ - } + {\text{H}} \cdot \to {{\text{H}}_2} + {\text{O}}{{\text{H}}^ - }$
$C_1^{14},{\text{ }}C_4^{14}$	$3.0 \times {10^{10}}$	${\text{e}^ - } + {\text{OH}} \cdot \to {\text{OH}^ - }$
$C_1^{{{14}^{^{\prime}}}},{\text{ }}C_4^{{{14}^{^{\prime}}}}$	$2.2 \times {10^{10}}$	${{\text{e}}^ - } + {{\text{O}}^ - } \cdot \to 2{\text{O}}{{\text{H}}^ - }$
$C_1^{1p},{\text{ }}C_5^{1p}$	$2.3 \times {10^{10}}$	${{\text{e}}^ - } + {{\text{H}}^ + } \to {\text{H}} \cdot $
$C_1^{13},{\text{ }}C_3^{13},{\text{ }}C_4^{13}$	$1.1 \times {10^{10}}$	${{\text{e}}^ - }{{\text{H}}_2}{{\text{O}}_2} \to {\text{OH}} \cdot + {\text{O}}{{\text{H}}^ - }$
$C_1^{{{13}^{^{\prime}}}},{\text{ }}C_3^{{{13}^{^{\prime}}}},{\text{ }}C_4^{{{13}^{^{\prime}}}}$	$3.5 \times {10^9}$	${{\text{e}}^ - } + {\text{HO}}_2^ - \to {\text{OH}} \cdot + 2{\text{O}}{{\text{H}}^ - }$
$C_1^{12},{\text{ }}C_2^{12},{\text{ }}C_7^{12}$	$1.9 \times {10^{10}}$	${{\text{e}}^ - } + {{\text{O}}_2} \to {\text{O}}_2^ - \cdot $
$C_1^{{{17}^{^{\prime}}}},{\text{ }}C_7^{{{17}^{^{\prime}}}}$	$1.3 \times {10^{10}}$	${{\text{e}}^ - } + {\text{O}}_2^ - \cdot \to {\text{O}}_2^{2 - }$
$C_1^{5h},{\text{ }}C_5^{5h}$	$2.2 \times {10^7}$	${\text{H}} \cdot + {\text{O}}{{\text{H}}^ - } \to {{\text{e}}^ - }$
$C_1^{1{\text{R}}}$	$1.4 \times {10^8}$	${\text{RH}} + {{\text{e}}^ - } \to {\text{R}}{{\text{H}}^ - }$
$C_2^{52},{\text{ }}C_5^{52},{\text{ }}C_7^{52}$	$2.1 \times {10^{10}}$	${\text{H}} \cdot + {{\text{O}}_2} \to {\text{H}}{{\text{O}}_2} \cdot $
$C_2^{{{47}^{^{\prime}}}},{\text{ }}C_4^{{{47}^{^{\prime}}}},{\text{ }}C_7^{{{47}^{^{\prime}}}}$	$8.0 \times {10^9}$	${\text{OH}} \cdot + {\text{O}}_2^ - \cdot \to {\text{O}}{{\text{H}}^ - } + {\text{O}}2{ }$
$C_2^{42},{\text{ }}C_4^{4^{^{\prime}}2}$	$3.6 \times {10^9}$	${{\text{O}}^ - } \cdot + {{\text{O}}_2} \to {\text{O}}_3^ - $
$C_2^{4^{^{\prime}}7^{^{\prime}}},{\text{ }}C_4^{4^{^{\prime}}7^{^{\prime}}},{\text{ }}C_7^{4^{^{\prime}}7^{^{\prime}}}$	$6.0 \times {10^8}$	${{\text{O}}^ - } \cdot + {\text{O}}_2^ - \cdot \to 2{\text{O}}{{\text{H}}^ - } + {{\text{O}}_2}$
$C_2^{7^{^{\prime}}7^{^{\prime}}},{\text{ }}C_3^{7^{^{\prime}}7^{^{\prime}}},{\text{ }}C_7^{7^{^{\prime}}7^{^{\prime}}}/2$	$2 \times {10^9}$	$2{{\text{H}}^ + } + 2{\text{O}}_2^ - \cdot \to {{\text{H}}_2}{{\text{O}}_2} + {{\text{O}}_2}$
$C_2^{28},{\text{ }}C_8^{28},{\text{ }}C_9^{28}$	$5 \times {10^7}$	${\text{R}} \cdot + {{\text{O}}_2} \to {\text{R}}{{\text{O}}_2} \cdot $
$C_2^{99}$	$1.0 \times {10^4}$	$2{\text{ROO}} \cdot \to {{\text{O}}_2} + {\text{ROH}} + {\text{RO}}$
$*C_2^{3w},{\text{ }}C_3^{2w}/2$	$6.62 \times {10^7}{{\text{s}}^{ - 1}}$	$2{{\text{H}}_2}{{\text{O}}_2} \to {{\text{O}}_2} + 2{{\text{H}}_2}{\text{O}}$
$C_3^{53},{\text{ }}C_4^{53},{\text{ }}C_5^{53}$	$9.0 \times {10^7}$	${\text{H}} \cdot + {{\text{H}}_2}{{\text{O}}_2} \to {\text{OH}} \cdot + {{\text{H}}_2}{\text{O}}$
$C_3^{57},{\text{ }}C_5^{57},{\text{ }}C_7^{57}$	$1.0 \times {10^{10}}$	${\text{H}} \cdot + {\text{H}}{{\text{O}}_2} \cdot \to {{\text{H}}_2}{{\text{O}}_2}$
$C_3^{44},{\text{ }}C_4^{44}/2$	$\frac{{1.1}}{2} \times {10^{10}}$	$2{\text{OH}} \cdot \to {{\text{H}}_2}{{\text{O}}_2}$
$C_3^{{{43}^{^{\prime}}}},{\text{ }}C_4^{{{43}^{^{\prime}}}},{\text{ }}C_7^{{{43}^{^{\prime}}}}$	$7.5 \times {10^9}$	${\text{OH}} \cdot + {\text{HO}}_2^ - \to {\text{O}}{{\text{H}}^ - } + {\text{H}}{{\text{O}}_2} \cdot &lt; &gt; {\text{O}}_2^ - \cdot + {{\text{H}}^ + }$
$C_3^{3I},{\text{ }}C_4^{3I}$	$1.0 \times {10^3}$	${\text{F}}{{\text{e}}^{2 + }} + {{\text{H}}_2}{{\text{O}}_2} \to {\text{F}}{{\text{e}}^{3 + }} + {\text{O}}{{\text{H}}^ - } + {\text{HO}} \cdot $
$C_4^{5w},{\text{ }}C_5^{5w},{\text{ }}C_6^{5w}$	$1.0 \times {10^1}$	${\text{H}} \cdot + {{\text{H}}_2}{\text{O}} \to {{\text{H}}_2} + {\text{OH}} \cdot $
$C_4^{54},{\text{ }}C_5^{54}$	$7 \times {10^9}$	${\text{H}} \cdot + {\text{OH}} \cdot \to {{\text{H}}_2}{\text{O}}$
$C_4^{46},{\text{ }}C_5^{46}$	$4.2 \times {10^7}$	${\text{OH}} \cdot + {{\text{H}}_2} \to {\text{H}} \cdot + {{\text{H}}_2}{\text{O}}$
$C_4^{4h}$	$1.3 \times {10^{10}}$	${\text{OH}} \cdot + {\text{O}}{{\text{H}}^ - } \to {{\text{O}}^ - } \cdot + {{\text{H}}_2}{\text{O}}$
$C_4^{4^{^{\prime}} w}$	$1.8 \times {10^6}$	${{\text{O}}^ - } \cdot + {{\text{H}}_2}{\text{O}} \to {\text{O}}{{\text{H}}^ - } + {\text{OH}} \cdot $
$C_4^{4{\text{R}}},{ }C_8^{4{\text{R}}}$	$1.0 \times {10^9}$	${\text{OH}} \cdot + {\text{RH}} \to {{\text{H}}_2}{\text{O}} + {\text{R}} \cdot $
$C_4^{4G}$	$1.0 \times {10^{10}}$	${\text{OH}} \cdot + {\text{GSH}} \to {{\text{H}}_2}{\text{O}} + {\text{GS}} \cdot $
$C_5^{55},{\text{ }}2C_6^{55}$	$1.55 \times 10^{\wedge}10$	${\text{H}} \cdot + {\text{H}} \cdot \to {{\text{H}}_2}$
$C_5^{5{\text{R}}}$	$1.0 \times {10^8}$	${\text{RH}} + {\text{H}} \cdot \to {\text{R}}{{\text{H}}_2} \cdot $
$C_6^{64}$	$4.2 \times {10^7}$	${\text{OH}} \cdot + {{\text{H}}_2} \to {\text{H}} \cdot + {{\text{H}}_2}{\text{O}}$
$C_8^{9{\text{L}}},{ }C_9^{9{\text{L}}}$	$2.0 \times 10$	${\text{ROO}} \cdot + {\text{LH}} \to {\text{ROOH}} + {\text{L}} \cdot $
$*C_8^8$	$300\,{{\text{s}}^{ - 1}}$	Decay constant of ${\text{R}} \cdot $
$C_8^{88}$	$5 \times {10^7}$	$2{\text{R}} \cdot \to {\text{R}} - {\text{R}}$
$*C_9^9$	$\left( {0.65 + 0.0408} \right){{\text{s}}^{ - 1}}$	Decay constant of ${\text{ROO}} \cdot $
$C_9^{99}$	$2 \times {10^4}$	$2{\text{ROO}} \cdot \to {{\text{O}}_2} + {\text{ROH}} + {\text{RO}}$

Lipid peroxidation of phospholipids in cell may lead to cell signaling alternation, cell dysfunction and cell death (Jurkiewicz *et al* 2012, Dixon and Stockwell 2019) and has been proposed as one of the main indications for FLASH effects (Froidevaux *et al* 2023, Grilj *et al* 2024). In our study we used the production of ROOH, which is produced during chain propagation and at chain termination to represent the extent of lipid peroxidation, to study the effects from beam deliveries and tissue conditions.

In this study we modeled the example of skin irradiation FLASH effects following several FLASH preclinical and clinical studies (Vozenin *et al* 2019, Velalopoulou *et al* 2021, Gaide *et al* 2022, Singers Sørensen *et al* 2022, Mascia *et al* 2023b). Skin oxygenation varies between the epidermis, dermis, and follicular regions but all regions tend to be slightly hypoxic, and we chose $10\,\mu {\text{M}}$ ${{\text{O}}_2}$ as a representative value (Hendry *et al* 1982, Evans *et al* 2006). GSH is present in quite large quantities in many cells, in levels significantly higher than physiological oxygen concentrations (Pizzorno 2014). In skin GSH levels fluctuate between layers, and we chose an average of $410\,\mu {\text{M}}$ as a representative concentration (Engin 1976, Telorack *et al* 2016).

### 2.2. Simulation and validation with preclinical data

The heterogeneous temporal dose structure requires sufficiently fine time steps in solving the stiff system of the physicochemical model in equation (1) for a convergent solution. The Rodas5P stiff ODE solver in the high-performance computing language Julia (Bezanson *et al* 2017) was used to solve equation (1), leveraging its auto-differentiation capabilities for rapid and accurate Jacobian calculation. Additionally, the solver dynamically adjusted the time step size to accurately account for the time-dependent products from the chemical reactions. The simulations were terminated $150$ s after the field irradiation, at which point the system had reached equilibrium. The time evolution profiles of each species were tracked throughout the simulation.

Our previous study (Tan *et al* 2023) has validated the model with experimental data including the *in-vitro* oxygen reduction *G*-value (Cao *et al* 2021, El Khatib *et al* 2022), mice memory preservation FLASH dose rate threshold (Montay-Gruel *et al* 2017) and leg contracture and cytokines (Cunningham *et al* 2021) correlated with the modeled AUC of ${\text{ROO}} \cdot $. In this study, we additionally modeled the production of ROOH under electron FLASH and CONV beams to compare with the measured lipid peroxidation in the work of Froidevaux *et al* (2023). We also modeled the ROOH product under FLASH and CONV scattered proton beam irradiations to correlate with the mice skin toxicity scores (Velalopoulou *et al* 2021).

### 2.3. Voxel dose delivery from PBS

**Figure 1.**
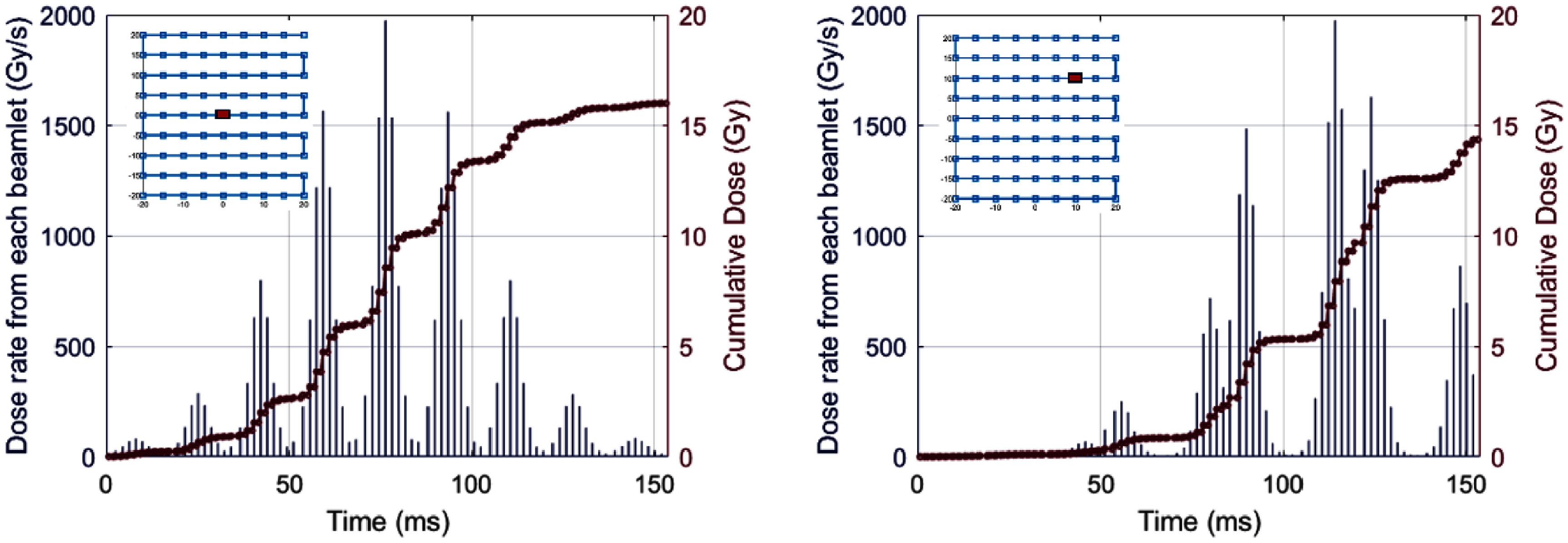
Varying instantaneous dose rate and cumulative dose structures at the Bragg peak depth from a ${ }40\, \times \,40\,{\text{m}}{{\text{m}}^2}{ }$ PBS field with ${ }5{\text{ mm}}$ spaced 227 MeV beam spots. The left figure shows for a point at the center of the field, while the right side shows the structures at a mid-diagonal point; the voxel position relative to the entire spot scanning pattern is shown in the top left inserts.

Our study considered the shoot-through UHDR PBS proton irradiation with maximum energy beam of 227 MeV and high beam current from a cyclotron-based accelerator. The target area such as in the case of skin irradiation was placed at the entrance plateau region to receive the desired irradiation dose. In a PBS delivery when the spots are delivered in a scanned grid pattern such as the inserts in figure 1, voxels at various depths and lateral distances receive much different dose delivery time structures. The instantaneous dose rate $\dot D\left( t \right)$ in each voxel depends on its distance from the spot and spot current (Zou *et al* 2020). The cumulative dose to a voxel in the treatment field is due to the contribution from multiple monoenergetic pencil beam spots, which can be expressed as

\begin{align*}D\left( {x,{ }y,z} \right) = \sum\limits_i {K_i}\left( {x,y,z} \right) \cdot {\text{M}}{{\text{U}}_i}\end{align*}

where ${\text{M}}{{\text{U}}_i}$ is the number of monitor units of the $i{\text{th}}$ proton spot at beam energy $E$, and the calculated dose kernel ${K_i}\left( {x,y,z} \right)$ in a water-equivalent phantom is the dose to the voxel at point $\left( {x,y,z} \right)$ from the $i{\text{th}}$ proton spot. This spot dose calculation was performed using Eclipse Treatment Planning System (Varian, Palo Alto, CA) using the proton convolution superposition algorithm, which resulted in a dose kernel with a spot sigma of 2.38 mm at the target depth of 8 mm. Spot MU delivery time $\Delta t{\text{ }}$ was calibrated with a Faraday cup that measured a specific proton spot delivery pulse time under specific beam currents and ratioed to the spot MUs. During the spot delivery, $\dot D\left( t \right) = \frac{D}{{\Delta t}}$. The spot to spot transition time was set to $1{\text{ ms}}$ as a comparable timescale with a cyclotron-based proton PBS delivery setup. During the spot transition period, $\dot D\left( t \right) = 0.$ Uniform field dose was developed by translating the dose kernel across a raster pattern, using equal MU weighting for each spot in accordance with previous work (Zou *et al* 2020). The raster pattern for a $40\, \times \,40\,{\text{m}}{{\text{m}}^2}$ field with $5\,{\text{mm}}$ spacing is shown in the top-left insets in figure 1. Using these parameters, figure 1 highlights the impact of spatiotemporal beam spot scanning on the dose structure at different voxel locations—the mid-diagonal field voxel displaying a different temporal dose accumulation structure from that of the central axis voxel. In this study, we considered the target region of a single depth layer of 3 mm using $1\, \times \,1 \times 3\,{\text{m}}{{\text{m}}^{\text{3}}}$ voxel resolution at 8 mm depth to mimic the skin irradiation.

### 2.4. Impact of PBS delivery parameters using RVH

The proton PBS instantaneous dose rate and cumulative dose buildup in a voxel depends on delivery parameters including the delivered dose, field size, pencil beam spot spacing and current. For each voxel in the target region, the time evolution of the ROS species in this voxel was simulated using the unique instantaneous dose rate $\dot D\left( {x,y,z} \right)$ profile in this voxel. To quantify the radiochemical inhomogeneity in the region of interest, we define the RVH to describe the volumetric radiation ROS production. We used ROOH-volume histogram to indicate the quantity of lipid peroxidation which can act as direct indicator of the differential irradiation effect between CONV and FLASH deliveries. We studied the impact of RVH from PBS doses of 2–15 Gy, field sizes of 40 × 40 up to 100 × 100 ${\text{m}}{{\text{m}}^2}$, pencil beam spot spacings of 3 and 5 mm, and proton beam currents of 1–500 nA.

Published preclinical experiment results showed that multi-beam deliveries resulted in reduced and even abrogated FLASH skin-sparing effect (Mascia *et al* 2023b, Sørensen *et al* 2024). We simulated the skin ROOH production from PBS UHDR delivery of 30 Gy using spot current of 250 nA with multi-beams and varied the time interval between beams, mimicking the experimental work by Mascia *et al* (2023b).

### 2.5. Correlation of RVH with different PBSDR definitions

Under the same PBS irradiation, various proposed definitions of a delivery ‘dose rate’ can have very different values, up to multiple folds of differences (Deffet *et al* 2023). These proposed definitions include the ADR that is the voxel dose divided by the PBS field delivery time, the DADR and PBSDR. The DADR to voxel $i$ is defined as

\begin{equation*}{\text{DAD}}{{\text{R}}_i} = \frac{1}{{{D_i}}}\sum\limits_j \left( {{d_{ij}}} \right)*\left( {{{\dot d}_{ij}}} \right)\end{equation*}

where ${D_i}$ is the total dose to the voxel, ${d_{ij}}$ and ${\dot d_{ij}}$ is the local dose and instantaneous local dose rate to voxel $i$ from spot $j$ delivery (van de Water *et al* 2019). The definition of PBSDR is

\begin{equation*}{\text{PBSD}}{{\text{R}}_i} = \frac{{{D_i} - 2{d^\dagger }}}{{{T_i}}}\end{equation*}

where ${d^\dagger } = 0.01\, \times \,D$ is 1% of the prescribed dose, and ${T_i}$ is the interval between reaching ${d^\dagger }$ and $D - {d^\dagger }$ (Folkerts *et al* 2020). We studied both the ROOH yields and the defined ‘dose rates’ in the voxels under various beam currents. We compared these ROOH yields to the one from uniform scattered proton beam delivery with 100 Gy s^−1^.

## 3. Results

**Figure 2.**
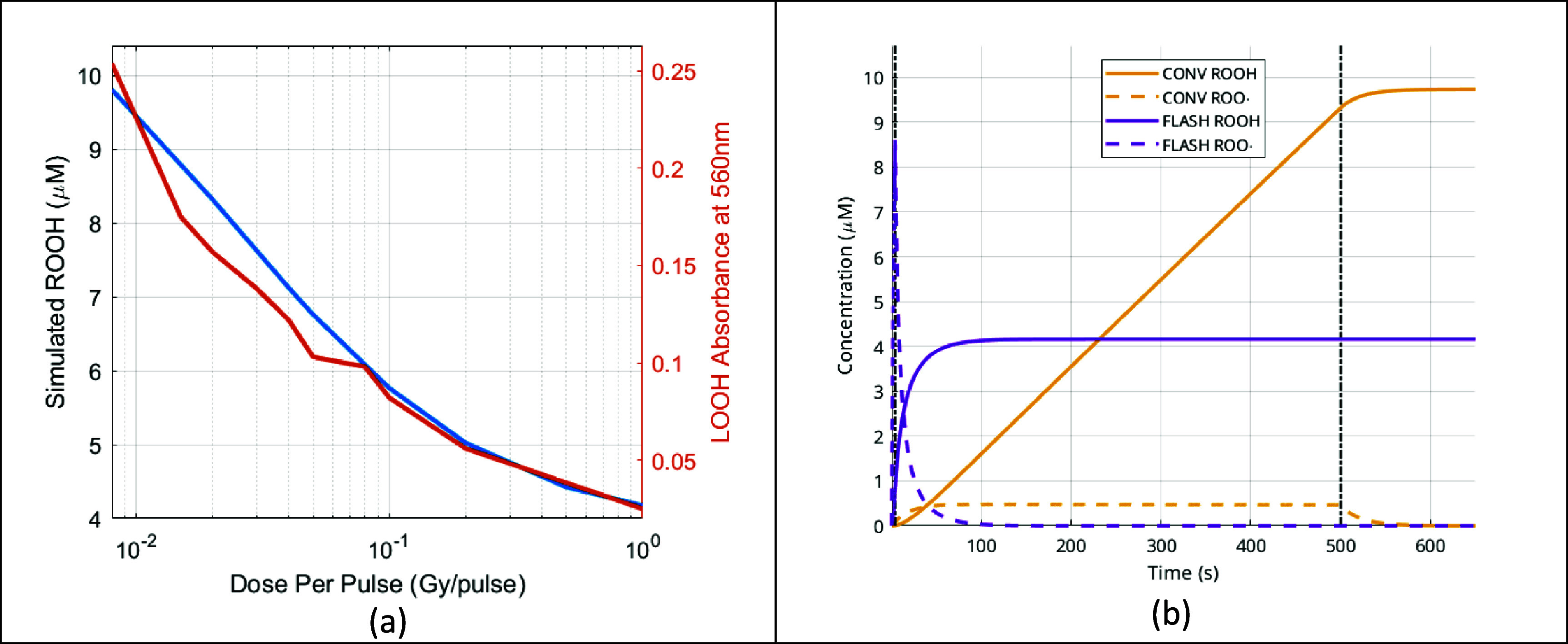
(a) A comparison of the simulated yield of alkyl hydroperoxide (blue) and measured LOOH absorbance values at 560 nm (orange) for lipid micelles following electron irradiation. (b) The evolution of ROOH and ${\text{ROO}} \cdot $ for 0.009 Gy/pulse (CONV) or 1 Gy/pulse (FLASH), with black vertical dashed lines indicating the beam-off times.

Our simulated ROOH concentrations from an electron beam at 10 Hz at various dose per pulse (DPP) to deliver a total of 40 Gy is shown in blue in figure 2(a). In accordance with the experimental setup, each pulse had a 1 $\mu {\text{s}}$ duration. As shown, the simulated ROOH decrease as a function of the logarithmically scaled DPP closely corresponds with LOOH absorbance measurements obtained from spectrophotometry by Froidevaux *et al* (2023). Our simulation mimicked the experiment setup using an ambient initial oxygen ${{\text{O}}_2}$ of 21% atm and fixing GSH to zero, as the experiment had no radical scavengers. Figure 2(b) shows the evolution of alkyl hydroperoxide and its radical, confirming that the system stabilizes within the allotted 150 s duration after beam off for both CONV and FLASH deliveries; for this plot the FLASH simulation was extended to end at the same time as the CONV simulation for easier comparison. In our simulation of the mice hind-leg skin from scattered proton irradiations with 30 and 45 Gy by Velalopoulou *et al* (2021), the ROOH productions for CONV 30, 45 Gy and FLASH 30, 45 Gy were 4.42, 6.14, 3.51 and 4.35 $\mu {\text{M}}$. These values correlate well with the skin toxicity data where the average skin damage scores of a cohort of 10 mice in each irradiation condition were 2.18, 2.58, 1.48 and 2.45. Note here the skin damage score for FLASH 45 Gy is close but slightly lower than for CONV 30 Gy, which is reflected from our simulated ROOH productions.

**Figure 3.**
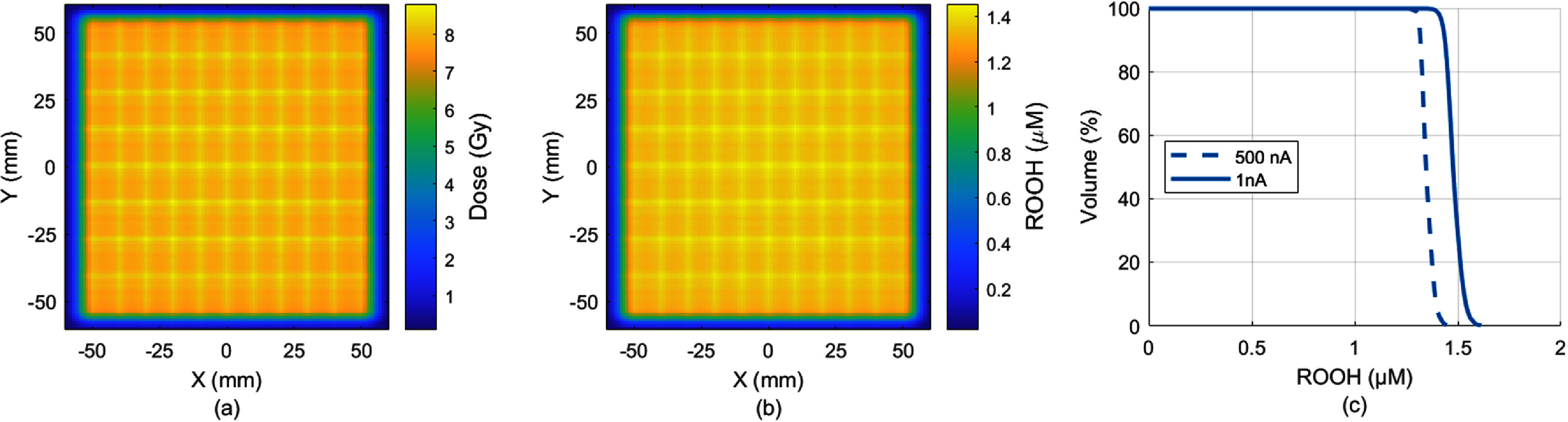
(a) The dose distribution from a nominal 8 Gy delivery to a 100 × 100 mm^2^ field at 8 mm depth with 3 mm spaced spots; (b) the ROOH yield when delivered with 500 nA FLASH pencil beam current; (c) the RVH curves under CONV 1 nA and UHDR 500 nA PBS deliveries.

Variations in pencil beam currents resulted in different delivery time with the same dose delivery and lead to differences in ROOH levels and ROOH-volume histograms. Figures 3(a) and (b) showed similarly patterned dose and ROOH color maps at 8 mm depth from a nominal 8 Gy PBS $100\, \times \,100{\text{ m}}{{\text{m}}^2}$ field with 3 mm spaced 227 MeV spots. The $100\, \times 100\, \times 3\,{\text{m}}{{\text{m}}^3}{ }$ region at the 8 mm depth with an average of 8 Gy was used for the RVH calculation. The ROOH-volume histogram in figure 3(c) clearly showed the reduction in ROOH due to increased pencil beam current from 1 nA to 500 nA, indicating potentially less cell damage. The mean ROOH for UHDR and CONV delivery over the region was 1.350 and 1.480 $\mu {\text{M}}$ with 0.130 $\mu {\text{M}}$ reduction due to FLASH dose rate. The 50% of the region volume ${\text{ROOH}}\left[ {50\% } \right]$ was 1.346 and 1.476 $\mu {\text{M}}$ indicating a reduction ${{\Delta {\text{ROOH}}}}\left[ {50\% } \right]{ }$ of 0.13 $\mu {\text{M}}$ due to the UHDR. Here we define the difference in ROOH produced at CONV vs UHDR for at least 50% of the volume as ${{\Delta {\text{ROOH}}}}\left[ {50\% } \right] = {\text{ROOH}}{\left[ {50\% } \right]_{500\,{\text{nA}}}}$ $- {\text{ROOH}}{\left[ {50\% } \right]_{1\,{\text{nA}}}}$. The subscript of $500{\text{ nA}}$ and $1{\text{ nA}}$ can also be replaced by other pencil beam currents used in PBS deliveries.

**Figure 4.**
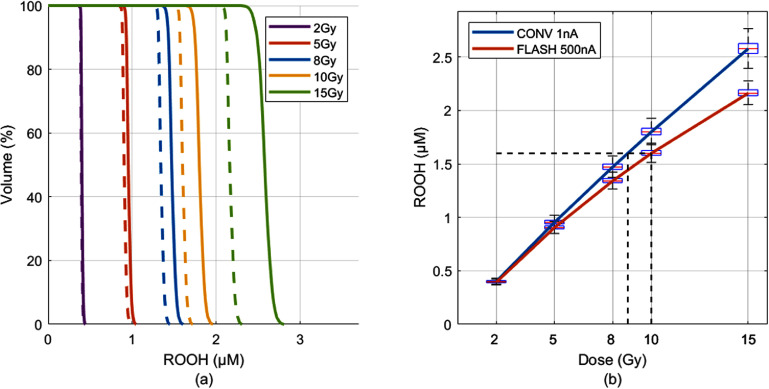
Effects of field dose on the RVH for a $100\, \times 100{\text{ m}}{{\text{m}}^2}$ field with 3 mm spacing. (a) The RVH curves for different doses, with dotted lines showing UHDR deliveries and solid lines showing CONV deliveries. (b) The ROOH with quartile values for the entire field vary with the dose. The solid lines are fitted curves for CONV and UHDR deliveries. The dash lines indicate one pair of CONV and UHDR dose that generate the same level of ROOH.

For various dose at 2, 5, 8, 10, and 15 ${\text{ Gy}}$ delivered to a $100\, \times 100{\text{ m}}{{\text{m}}^2}$ field with $3{\text{ mm}}$ spot spacing, the proton beam current was set at either 1 nA (CONV) or 500 nA (UHDR), and the RVH curves were derived for each pair of CONV and UHDR deliveries (figure 4(a)). In figure 4(a), higher doses produced larger ROOH values, indicating more radiation-induced tissue damage. Meanwhile, the reduction in ROOH from CONV to UHDR at the same dose also increased with the dose value, indicating potentially larger FLASH effect under higher dose. At low nominal doses of 2 and 5 Gy, ${{\Delta {\text{ROOH}}}}\left[ {50\% } \right]{ }$ was only $0.007{ }\mu {\text{M }}$ and $0.049{ }\mu {\text{M}}$, respectively. Increasing the doses to 8 Gy, 10 Gy, or 15 Gy raised the ${{\Delta {\text{ROOH}}}}\left[ {50\% } \right]$ to $0.130{ }\mu {\text{M}},{ }0.200{ }\mu {\text{M}},{ }0.417{ }\mu {\text{M}}$ showing more significant reduction in the ROOH from higher dose rate. Figure 4(b) showed that the field ROOH changed with field nominal dose, which can be used to determine the equivalent CONV dose and UHDR dose to create the same ROOH level. For example, the dashed lines indicated UHDR 10 Gy and CONV 8.78 Gy generated the same mean ROOH of $1.599{ }\mu {\text{M}}$ in the field, indicating a FLASH dose modifying factor 1.138. This FLASH dose modifying factor increases with increased dose, reaching 1.212 for UHDR 15 Gy delivery.

**Figure 5.**
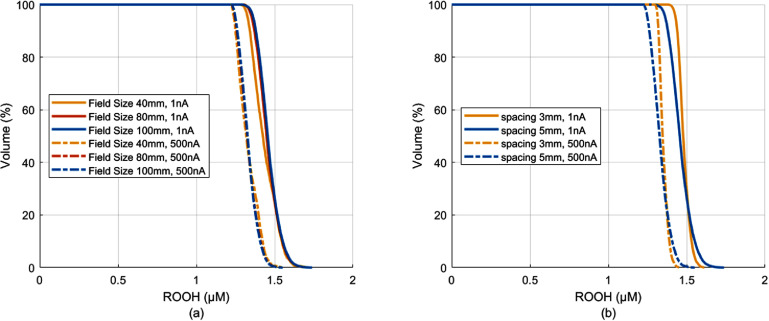
(a) RVHs of nominal 8 Gy delivery with 5 mm spaced spots to various field sizes show that ROOH yield relies on the pencil beam current but not on the field size; (b) under the same nominal dose 8 Gy to a field of $100\, \times 100{\text{ m}}{{\text{m}}^2}$, spot spacing has very small impact to ROOH-volume histograms.

The effects of PBS field size and spot spacing on ROS production were investigated. For PBS delivery of a nominal 8 Gy but with varied field sizes of $40 \times 40,{\text{ }}80 \times 80,{\text{ and }}100 \times 100{\text{ m}}{{\text{m}}^2}$, the ROOH-volume histograms for the in-field voxels were derived (figure 5(a)). The field delivery time increased with the field size, with 222.4 ms for $40 \times 40{\text{ m}}{{\text{m}}^2}$ as opposed to 1194.4 ms for $100 \times 100{\text{ m}}{{\text{m}}^2}$ for 500 nA pencil beams, due to the number of spots in the field. Although the traditional field ADR values decreased sharply from 35.97 Gy s^−1^ to 6.70 Gy s^−1^, surprisingly, there was minimal impact to the RVH curves (figure 5(a)). Figure 5(a) also showed very close RVHs for different field sizes for the CONV beam current of 1 nA. Increasing the beam current from 1 nA to 500 nA reduced the ROOH production by similar amount, with $\Delta {\text{ROOH}}\left[ {50\% } \right]{ }$ of 0.106, 0.123, and 0.130 $\mu {\text{M}}$ for $40 \times 40,{\text{ }}80 \times 80,{\text{ and }}100 \times 100{\text{ m}}{{\text{m}}^2},$ respectively. This result indicated that FLASH effect could be attainable over larger field, as long as the pencil beam current is sufficiently high.

The impact of pencil beam spot spacing on RVH was also examined using a 100 × 100 mm^2^ field with a nominal dose of $8{\text{ Gy}}$ at 3 mm and 5 mm spot spacings. The delivery time was 1760.3 ms with 1089 spots for the 3 mm spacing, and 1194.4 ms with 441 spots for the 5 mm spot spacing. Notably, the 3 mm spaced spots resulted in a more homogeneous dose and ROOH distributions. Figure 5(b) showed that the regional RVHs of the same beam current closely track each other, despite the differences in the spot spacing and field delivery time. The larger variations in ROOH values for 5 mm spaced spot delivery were attributed to the greater variability in voxel doses.

**Figure 6.**
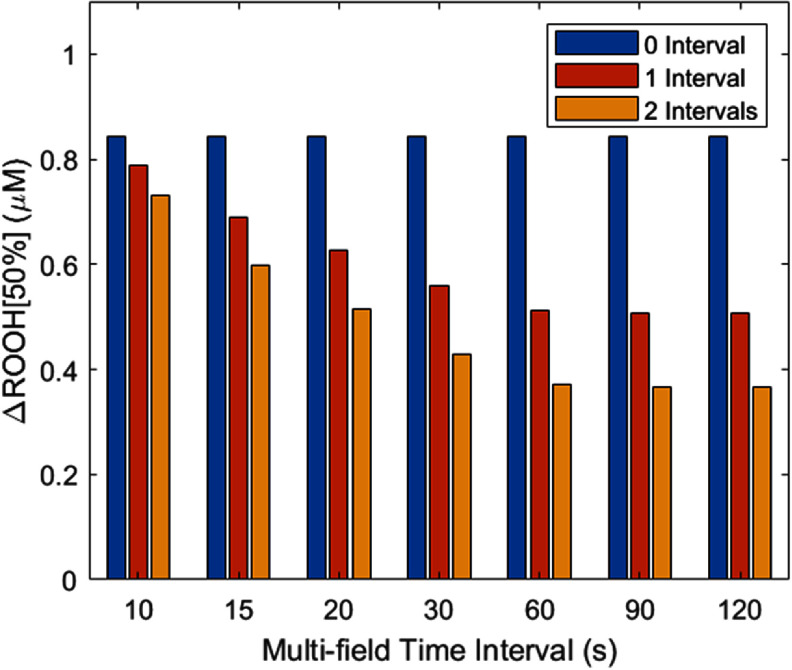
UHDR-induced ΔROOH[50%] from 1 nA to 250 nA 30 Gy PBS deliveries with no interval, 1 and 2 time intervals of various interval durations. The no interval bar was plotted at each interval length for better comparison.

We simulated the multi-field delivery mice experiments reported by Mascia *et al*, in which a 30 Gy dose was separated into 1, 2, or 3 deliveries with 2 min intervals between. We used 5 mm spaced spots to deliver 30 Gy to a $4 \times 4{\text{ c}}{{\text{m}}^2}$ field at 8 mm depth. For a continuous single delivery of 30 Gy, the ${\text{ROOH}}\left[ {50\% } \right]$ reduced from 4.224 $\mu {\text{M}}$ at 1 nA to 3.380 $\mu {\text{M}}$ at 250 nA with a ${{\Delta {\text{ROOH}}}}\left[ {50\% } \right] = 0.844{ }\mu {\text{M}}$. Introducing one 2 min time interval for 2 × 15 Gy delivery reduces the ${{\Delta {\text{ROOH}}}}\left[ {50\% } \right]$ to $0.507{ }\mu {\text{M}}$, indicating less sparing of the radiation damage. Two 2 min time intervals for $3\, \times 10\,{\text{Gy}}$ delivery resulted in a ${{\Delta {\text{ROOH}}}}\left[ {50\% } \right] =$ $0.366{ }\mu {\text{M}}$ indicating further reduced UHDR sparing. The decrease in ${{\Delta {\text{ROOH}}}}\left[ {50\% } \right]$ from multi-field delivery is consistent with the reduced FLASH effect observed by Mascia *et al.* We additionally found the length of the time interval affected the UHDR-induced reduction in ROOH, as shown in figure 6. UHDR-induced ${{\Delta {\text{ROOH}}}}\left[ {50\% } \right]$ decreases with increased time interval, until the time interval is above 60 s where the ${{\Delta {\text{ROOH}}}}\left[ {50\% } \right]$ plateaus, showing much lower ROOH reduction and reduced FLASH sparing. Based on this simulation, a time interval of 10 s or less maintains ∼90% of UHDR-induced ROS reduction, although such time intervals between the beams might not be clinically feasible. When the time interval is 60 s or longer, reduced or abrogated FLASH effect would be consistently observed.

**Figure 7.**
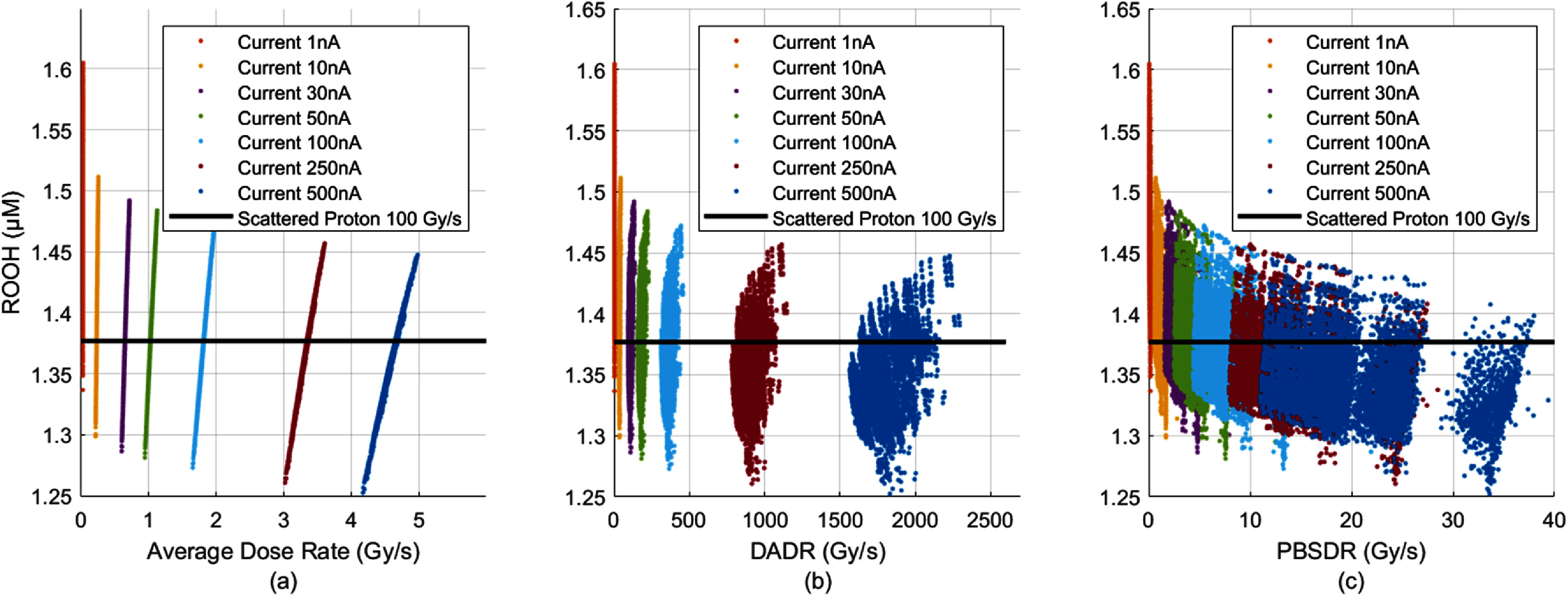
ROOH decreases in a similar asymptotic trend with three definitions of dose rate in the context of PBS delivery, for 8 Gy $100\, \times 100{\text{ m}}{{\text{m}}^2}$ delivery. Results from using multiple proton pencil beam currents are plotted.

To elucidate the relationship between various PBSDR definitions and ROOH reduction, we simulated 8 Gy $100\, \times 100{\text{ m}}{{\text{m}}^2}$ field with 3 mm spot spacing over a range of proton pencil beam currents from CONV 1 nA up to UHDR 500 nA. As expected, the field voxels at 8 mm depth had quite different ‘dose rate’ values from specific dose rate definitions and also relied on the spot scanning sequences (figure 7). However, all three dose rates (ADR, DADR and PBSDR) increase with increasing pencil beam currents. Moreover, an asymptotic reduction in ROOH with increasing proton current was observed. There is no apparent correlation between either of the three types of dose rates to the ROOH values.

For a preclinically validated uniform scattered 100 Gy s^−1^ proton field demonstrated to FLASH produce FLASH effects (Diffenderfer *et al* 2020) the predicted ROOH yield is 1.377 $\mu {\text{M}}$. Using this value as a threshold, we calculated that increasing the proton current from 1 nA to 250 nA would increase the percentage of voxels under the threshold from 0.3% to 80.2%, indicating that a majority of the field would experience FLASH effects at a beam current of 250 nA. Further increasing in the proton current to 500 nA resulted in a slight increase in the number of voxels (86.1%) below the ROOH threshold level, indicating no drastic change in FLASH sparing observations. At 8 mm depth ${\text{ROOH}}\left[ {50\% } \right]{ }$ was 1.470, 1.359, 1.347 and 1.340 $\mu {\text{M}}$ for proton currents of 1, 100, 250 and 500 nA, respectively, indicating a drastic drop in ROOH from 1 to 100 nA pencil beam that plateaus with higher beam current.

## 4. Discussion

Our study presented the first radiochemical simulation of proton PBS CONV and UHDR delivery, incorporating spot spacing and PBS delivery timing structure to study the relationship of the ROS production and field delivery parameters. We introduced the RVH in the region of interest as a useful index to quantify the ROS reduction from UHDR. We showed that delivered total dose and proton pencil beam current were the key parameters for UHDR-induced ROOH reduction from ROOH produced under CONV delivery, but the PBS field size or spot spacing had minimal impact. This finding has an important indication in FLASH studies that despite the largely varying dose rate values from multiple definitions and with the field size and spot spacing (van de Water *et al* 2019, Folkerts *et al* 2020, Zou *et al* 2020, Deffet *et al* 2023), the FLASH effect is likely caused mostly by the delivered dose and scanned pencil beam current, and relatively independent of treatment field size and spot spacing. Therefore FLASH effects can potentially be observed from large PBS scanning field with sufficiently high pencil beam current. This is especially beneficial for translational patient FLASH radiotherapy to treat large tumor targets.

The amount of ROOH reduction from CONV to UHDR delivery depended on the total prescribed dose. Such dose dependent behavior is consistent with the hypothesis that under UHDR delivery, larger initial deposition of radicals leads to higher intra-radical recombination, since a minimal deposition would be required before such second-order kinetics were achieved. This suggests that FLASH delivery must attain a baseline dose to exhibit sparing due to a ROS reduction threshold, which may lend itself well to hypofractionation.

Adequate and homogeneous dose coverage in the radiation treatment delivery can use smaller spot spacing relative to the spot size. On the other hand, FLASH delivery under UHDR demands shorter field delivery time with less spots to avoid excessive spot-to-spot transition time. In our study, we chose the spot spacings that still maintain very reasonable uniform field dose distribution—D95% is 7.74 and 7.31 Gy for the fields with 3 and 5 mm spacing, respectively, while maintaining the mean field dose as 8.00 Gy. However, caution must be taken when too large a spot spacing is used that creates heterogeneous dose in the field. The variations in voxel dose then become a confounding factor for the dose rate effect and ROOH sparing when comparing the CONV to FLASH deliveries.

Another concern in translating FLASH from preclinical to patient treatment is the multi-beam delivery in typical clinical patient treatment. Studies have indicated that the time intervals between beams can reduce FLASH sparing in mice skin irradiations (Mascia *et al* 2023b, Sørensen *et al* 2024). Our simulation results were consistent with these data, showing much smaller ROOH reduction with time intervals longer than 60 s. We additionally pointed out shorter time interval still has a chance to maintain the FLASH effect, for example, the time interval of 10 s maintains ∼90% of ROS reduction, although such time intervals between the beams might not be clinically feasible. Overall, these results underscore the need for effective FLASH UHDR delivery with minimal beam disruptions.

Our findings that FLASH causes changes in lipid peroxidation is generally consistent with new findings that FLASH reprograms lipid expression in macrophages (Ni *et al* 2025). The differential levels of oxylipins under CONV and FLASH irradiations were also demonstrated experimentally (Portier *et al* 2024). Our model does not explore products downstream of ROOH, though it is quite likely that these products could be transformed into immunogenic eicosanoids or other oxylipins over longer time scales (Brash 2001, Ayala *et al* 2014). Tracking the production of species downstream of ROOH requires further understanding of the processes and associated reaction rate constants on the multiplicity of possible endpoints, as poly-unsaturated fatty acids have multiple bis-allylic carbons whose stereochemistry allows for nonspecific radical attacks (Furse *et al* 2006).

One limitation in our simulation approach was our assumption of the homogeneous system after using MC simulated physics processes and initial water radiolysis products. Monte Carlo code such as GEANT4-DNA and TOPAS-nBio (Incerti *et al* 2016, Schuemann *et al* 2019) were developed to integrate the physical–chemical and chemical processes after the physics of the local irradiation. It has since been adapted to several other packages for cellular-level radiobiology studies including the investigation of the FLASH track yields and radical–radical recombination (Lai *et al* 2020, Ramos-Méndez *et al* 2020, Boscolo *et al* 2021, D-Kondo *et al* 2023, Shin *et al* 2024). These MC codes have the ability to track the diffusion and reactions of individual species with inhomogeneous distribution. While MC approaches offer greater precision, simulations are computationally expensive and limited to very small volumes. Our approach, on the other hand, simulates each voxel at millimeter scale, assuming homogeneous concentration after irradiation and ignoring variations in the intracellular and extracellular environment. The simulation yields the ROS productions in much larger volume with reasonably fast speed. However, the impact of these assumptions could be further investigated.

## 5. Conclusion

Our study used a physicochemical metric ROOH and its associated ROOH-volume histogram in the region of interest to unify beam delivery parameters for proton UHDR PBS FLASH implementation while there is a lack of consensus on the appropriate dose rate definition to use. The ROOH metric corresponds to the lipid peroxidation damage from ROS production under different dose rates. Our study showed that ROOH reduction depends strongly on dose and pencil beam current, but much less to the field size and spot spacing. The ROOH yields showed asymptotic behavior with various dose rate definitions such as ADR, DADR, and PBSDR, with proton current being an important indicator of the ROOH values. We also studied the effect of extending the intervals between beams that has shown in *in-vivo* studies to reduce FLASH effect. Volumetric statistics using ROOH-volume histograms appear to serve as a signpost to aid the interpretation of the FLASH effects in an irradiated volume. This biology-driven metric has the potential to be used for correlation with the FLASH outcome studies.

## Data Availability

The data cannot be made publicly available upon publication because they are not available in a format that is sufficiently accessible or reusable by other researchers. The data that support the findings of this study are available upon reasonable request from the authors.
